# Mast Cell Infiltration and Subtype Promote Malignant Transformation of Oral Precancer and Progression of Oral Cancer

**DOI:** 10.1158/2767-9764.CRC-24-0169

**Published:** 2024-08-22

**Authors:** Xin-Jia Cai, Chao-Ran Peng, Jian-Yun Zhang, Xue-Fen Li, Xu Wang, Ying Han, He-Yu Zhang, Xin Peng, Tie-Jun Li

**Affiliations:** 1 Central Laboratory, Peking University School and Hospital of Stomatology, Beijing, China.; 2 Department of Oral Pathology, Peking University School and Hospital of Stomatology, Beijing, China.; 3 Department of Oral Medicine, Peking University School and Hospital of Stomatology, Beijing, China.; 4 Department of Oral and Maxillofacial Surgery, Peking University School and Hospital of Stomatology, Beijing, China.; 5 National Center for Stomatology, National Clinical Research Center for Oral Diseases, National Engineering Research Center of Oral Biomaterials and Digital Medical Devices, Beijing Key Laboratory of Digital Stomatology, Research Center of Engineering and Technology for Computerized Dentistry Ministry of Health, NMPA Key Laboratory for Dental Materials, National Engineering Research Center of Oral Biomaterials and Digital Medical Devices, Beijing, China.; 6 Research Unit of Precision Pathologic Diagnosis in Tumors of the Oral and Maxillofacial Regions, Chinese Academy of Medical Sciences (2019RU034), Beijing, China.

## Abstract

The role of mast cell (MC), a common myeloid-derived immune cell, in the development of oral squamous cell carcinoma (OSCC) is unclear. The aim of this study was to investigate MC infiltration in oral precancer and oral cancer. The evaluation of immune cell infiltration and its association with prognosis in OSCC used RNA sequencing and multiple public datasets. Multiplex immunofluorescence was used to explore the infiltration of MC in the microenvironment of OSCC and oral precancer and the interaction with CD8^+^ cells. The role of MC in OSCC progression was verified by *in vivo* experiments. The resting MC infiltration was mainly present in oral precancer, whereas activated MC infiltration was significantly higher in OSCC. Activated MC was associated with malignant transformation of oral precancer and poor prognosis of OSCC. *In vivo* studies showed that MC promoted the growth of OSCC. The infiltration of activated MC was negatively correlated with the infiltration of CD8^+^ T cells. The subtype of MC containing tryptase without chymase (MC_T_) was significantly higher in OSCC compared with oral precancer and was associated with poor survival. Furthermore, spatial distance analysis revealed a greater distance between MC_T_ and CD8^+^ cells, which was also linked to poor prognosis in OSCC. Cox regression analysis showed that MC_T_ could be a potential diagnostic and prognostic biomarker. This study provides new insights into the role of MC in the immune microenvironment of OSCC. It might enhance the immunotherapeutic efficacy of OSCC by developing targeted therapies against MC.

**Significance:**

In this study, we investigated the role of mast cells (MC) in oral precancer and oral cancer and demonstrated that MCs are involved in oral cancer progression and may serve as a potential diagnostic and prognostic marker. It might improve the immunotherapeutic efficacy through developing targeted therapies against MCs.

## Introduction

Head and neck squamous cell carcinomas arise from the mucosal epithelium of the oral cavity, pharynx, and larynx and are the most prevalent malignant tumors in the head and neck region, with the oral cavity being the most affected site (1). The consumption of tobacco, alcohol, and betel nut, as well as genetic factors, contribute to the development of oral squamous cell carcinoma (OSCC; ref. 1). The worldwide incidence cases of oral cancer rose from 185,976 in 1990 to 370,000 in 2019, whereas mortalities increased from 97,492 in 1990 to 199,000 in 2019 (2). The incidence of oral cancer rises with age and is considerably higher among males than females (3). A proportion of OSCC arises from the progression of oral precancer, but a considerable number of patients present with advanced OSCC at first diagnosis, resulting in a poor prognosis with a 5-year survival rate of 57.9% (4).

Oral leukoplakia (OLK) is a common precursor lesion of OSCC with a high malignant potential (5). Genetic variations and environmental factors may contribute to the aberrant proliferation, maturation, and differentiation of the epithelium in OLK, as well as alterations in immune cell infiltration in the stroma, which may be involved in malignant transformation (6, 7). The role of epithelial dysplasia in promoting the risk of malignant transformation of OLK has been elucidated (8). However, the role of immune cells, particularly myeloid cells, in the development from OLK to OSCC remains unclear.

The immune microenvironment (IME) represents a complicated ecosystem consisting of cellular and noncellular components in OSCC. The cellular components include infiltrating immune cells, such as T cells, B cells, NK cells, dendritic cells (DC), and macrophages, whereas the noncellular components comprise diverse cytokines and chemokines (9, 10). IME plays a vital role in tumor progression, metastasis, and therapy resistance (10). Targeted immunotherapy has improved patient survival in OSCC (11). However, fewer than 20% of patients exhibit a lasting response to these treatments (12). Therefore, in order to enhance the treatment and prognosis, developing new immunotherapies that address additional important factors in the complex IME of OSCC is essential (9).

Mast cells (MC) are myeloid tissue-resident immune cells. They have been implicated in the pathogenesis of allergic and autoimmune diseases. Recently, MCs have been identified as a promising but often overlooked target in cancer immunotherapy (13). The combination of MC inhibitors with antiprogrammed–cell death protein 1 (PD1) has been demonstrated to be an effective treatment for tumors, with the potential to overcome resistance to immune checkpoint inhibitors (14). The cytoplasm of MC contains basophilic granules with various bioactive substances, including histamine, leukotrienes, tryptase, chymase, and inflammatory factors. The phenotypic and functional heterogeneity of MC can be classified into subtypes depending on whether they contain tryptase and chymase (MC_TC_) or contain tryptase without chymase (MC_T_; refs. 13, 15). MC is located at the interface between the host and the environment, distributed closely to the basement membrane in normal oral mucosa, and can respond to the alterations in the surrounding microenvironment (16).

In this study, we investigated the contribution of MC to the development and progression of OSCC by examining immune cell infiltration and explored the variances in MC subtypes in oral precancer and OSCC to identify new ideas of targeted immunotherapy involving MCs.

## Materials and Methods

### Tissue sample collecting

Frozen samples of OLK (*n* = 11) and OSCC (*n* = 20) were prospectively collected for RNA sequencing (RNA-seq; Supplementary Table S1). The formalin-fixed, paraffin-embedded samples of OLK (*n* = 50) and OSCC (*n* = 50) were retrospectively collected for immunofluorescence staining (Supplementary Table S2). All OLK and OSCC samples were diagnosed and reviewed by two experienced pathologists according to the World Health Organization diagnostic criteria (17). The experimental procedures of this study were approved by the Institutional Ethics Committee of the school (PKUSSIRB202385022).

### Total RNA extraction and RNA sequencing

Total RNA was extracted and purified using TRIzol (Invitrogen) according to the manufacturer’s protocol. RNA-seq was performed by Beijing Novogene Technology Corporation. Libraries were constructed using the NEBNext Ultra RNA Library Prep Kit for Illumina according to the manufacturer’s protocol. Briefly, RNA integrity was assessed using the RNA Nano 6000 Assay Kit of the Bioanalyzer 2100 system (Agilent Technologies). To preferentially select cDNA fragments of 370 to 420 bp, library fragments were purified using the AMPure XP system. PCR products were purified (AMPure XP system), and library quality was assessed on the Agilent Bioanalyzer 2100 system. Library preparations were sequenced on an Illumina Novaseq platform and 150-bp paired-end reads were generated. The reference genome was indexed using Hisat2 v2.0.5 and the clean paired-end reads were aligned to the reference genome using Hisat2 v2.0.5. The featureCounts v1.5.0-p3 was used to count the number of reads mapped to each gene. The Fragments Per Kilobase of sequence per Million mapped reads of each gene were then calculated based on the length of the gene and the number of reads mapped to that gene. The human GRCh38 reference genome was used for the RNA-seq alignment.

### OLK and OSCC datasets acquisition

Two datasets (GSE85195 and GSE30784) containing OLK and OSCC gene expression were collected from the Gene Expression Omnibus (GEO) database (RRID: SCR_005012, https://www.ncbi.nlm.nih.gov/geo/) to compare the differences in immune cell infiltration in OLK and OSCC (18, 19). OLK dataset GSE26549 and OSCC dataset GSE41613 from the GEO database and the Head and Neck Cancer (HNSC) cohort of The Cancer Genome Atlas (TCGA) from UCSC Xena (RRID: SCR_018938, http://xena.ucsc.edu/) were used to analyze the relationship between MC infiltration and prognosis (20–22).

### Multiplexed immunofluorescence staining and analysis

As described previously (23), immunofluorescence staining was performed using the PANO 7-plex IHC kit, cat0004100100 (Panovue), and mouse antitryptase antibody (RRID: AB_303023, 1:200), rabbit antichymase antibody (Abcam, Ab186417, 1:500), mouse anti-CD8 antibody (RRID: AB_2799781, 1:200), and mouse antipan-CK antibody (RRID: AB_476839, 1:100) were applied sequentially, followed by horseradish peroxidase-conjugated secondary antibody incubation and tyramide signal amplification, slides were microwaved after each tyramide signal amplification procedure, and finally treated with 4′,6-diamidino-2-phenylindole (DAPI, SIGMA-ALDRICH) for nuclear visualization. Whole slide scanning of immunofluorescence images was performed using an Olympus VS200 MTL (Olympus Germany), and whole slide images were subjected to quantitative pathologic analysis using QuPath software (RRID: SCR_018257). The stromal area of the whole slide image was manually annotated by two experienced pathologists. The positive cell ratio was assessed based on the number of positive cells/total immune cells of the stromal area. Epithelial cells in OLK and carcinoma cells in OSCC were excluded by pan-CK staining. The intercellular distance data are all 2D Cartesian coordinate system data, and the default data are based on the pixel coordinates of the rectangular image field of view, which can be converted to the corresponding length coordinate data and calculated according to the magnification. The distance is calculated using the Euclidean distance.

$$d\left(x,y\right)∶=\sqrt{{\left(x_{1}-y_{1}\right)}^{2}+{\left(x_{2}-y_{2}\right)}^{2}+\cdots {+\left(x_{n}-y_{n}\right)}^{2}}=\sqrt{\overset{n}{\underset{i=1}{\sum }}{\left(x_{i}-y_{i}\right)}^{2}}$$

Because we are currently only dealing with two-dimensional coordinate data, this can be simplified to the following equation:

$$d=\sqrt{{\left(x_{2}-x_{1}\right)}^{2}+{\left(y_{2}-y_{1}\right)}^{2}}.$$

### Generation of bone marrow–derived MCs

Based on the report by Huang and colleagues (24), bone marrow cells were obtained from mice femurs and cultured in RPMI 1640 supplemented with 10% FBS, 2-mmol/L L-glutamine, 1-mmol/L sodium pyruvate, 1-mmol/L 4-(2-hydroxyethyl)-1-piperazineethanesulfonic acid, 50-μmol/L 2-ME, 100-U/mL penicillin, and 100-μg/mL streptomycin. The cells were cultured in the presence of 10-ng/mL rmIL3 (PeproTech, Cat# 213-13) and 20-ng/mL rmSCF (PeproTech, Cat# 250-03), and the nonadherent cells were passaged every 3 days. Four weeks later, the cells were used as MCs for experiments and identified as bone marrow–derived MCs (BMMCs).

### *In vivo* tumor growth experiment

C3H/HeJ mice (RRID: IMSR_JAX:000659) of 7 weeks old were purchased from Cyagen, and the procedure was performed in compliance with the regulations and the Peking University institutional animal care guidelines. The mouse squamous cell carcinoma cell line, SCC7 (RRID: CVCL_V412), was obtained from the Central Laboratory of Peking University School and Hospital of Stomatology and cultured according to their guidelines. C3H/HeJ mice were inoculated with a mixture of SCC7 tumor cells (10^5^ cells) and BMMCs (10^5^ cells) by subcutaneous injection to the dorsum of the tongue, whereas the control mice were inoculated with an equivalent mixture of SCC7 tumor cells and PBS. A week later, daily measuring of mouse weight and measuring of tumor growth every 2 days were conducted. All mice were sacrificed on the 17^th^ day for experiments. The tumor growth was monitored by measuring the length (*L*) and width (*W*) of tumors. The volume (*V*) of the tumor was calculated by the formula *V* = (*L* × *W*^2^)/2. All animal procedures were approved by Peking University Institutional Animal Care and Use Committee (No. LA2023281).

### Hematoxylin–eosin staining and IHC staining

After formaldehyde fixation, dehydration, and paraffin embedding, the tissues were cut into 4 μm sections and stained with hematoxylin and eosin (H&E) and IHC staining. The IHC staining was performed using the GTVisionTM III Detection System (Gene Tech, GK500710) according to the manufacturer’s protocol. The primary antibodies include rabbit anti-CD117 antibody (RRID: AB_2891166, 1:4,000), rabbit anti-Ki67 (RRID: AB_302459, 1:200), rabbit anticytokeratin (CK, RRID: AB_307222, 1:100) and rabbit anti-CD8 (RRID: AB_2860566, 1:1,000). As described previously (25), three positive staining areas were imaged at ×200 magnification. Positive cells in each image were counted by ImageJ version 1.53 (RRID:SCR_003070), and the average number of cells was considered to be the final staining evaluation.

### Bioinformatics and statistical analysis

The CIBERSORT analysis tool (RRID: SCR_016955, https://cibersort.stanford.edu/) was used to determine the abundance of immune cells with reference to the LM22 signature and 1,000 permutations. The CIBERSORT deconvolution algorithm was relatively accurate and robust and was validated to estimate the proportions of 22 types of immune cells such as B cells, T cells, macrophages, DCs, and MCs from microarray expression data (26). Mann–Whitney test and Welch’s *t* test were used to compare data between the two groups. Cox regression analysis was used to investigate the relationship between risk factors and prognosis. Kaplan–Meier (KM) curves were used for prognostic comparisons. *P* < 0.05 was considered statistically significant.

### Data availability

The RNA-seq data of OLK and OSCC were available to the NCBI GEO database (https://www.ncbi.nlm.nih.gov/geo/) and the TCGA database (https://portal.gdc.cancer.gov/). The RNA-seq data of the samples used in this study have been uploaded to the Genome Sequence Archive-Human Database of the National Genomics Data Center (subHRA010828, https://ngdc.cncb.ac.cn/gsa-human/).

## Results

### Differences in immune cell infiltration between oral precancer and oral cancer

**Figure 1:**
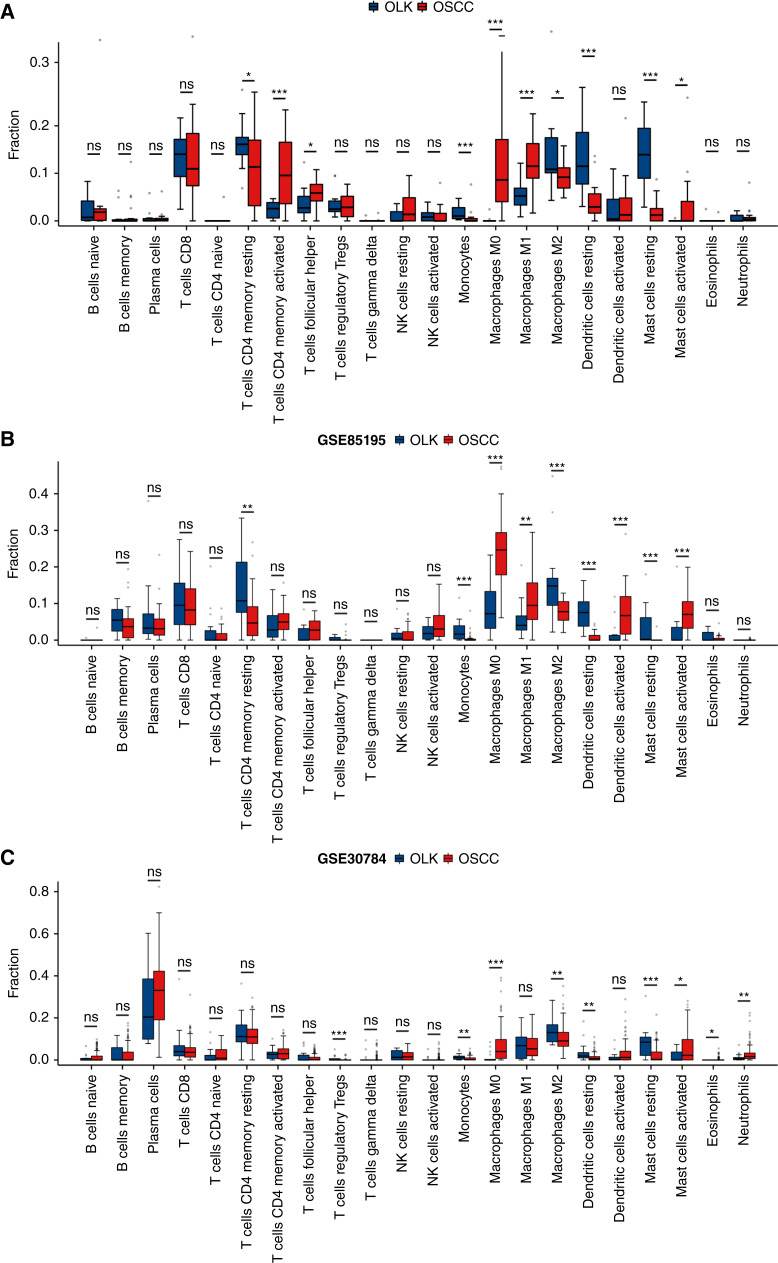
Differences of relative fraction in immune cell infiltration between oral precancer and OSCC using the CIBERSORT deconvolution algorithm for immune cell infiltration analysis in RNA-seq of 31 cases of frozen samples **A,** and two datasets from the GEO database (**B,** GSE85195; **C,** GSE30784). *, *P* < 0.05; **, *P* < 0.01; ***, *P* < 0.001; ns, not significant.

Using the CIBERSORT deconvolution algorithm for immune cell infiltration analysis in the RNA-seq data of our collected samples, it was found that the infiltration of resting CD4 T cells (*P* = 0.039), monocytes (*P* < 0.001), M2 macrophages (*P* = 0.030), resting DCs (*P* < 0.001), and resting MCs (*P* < 0.001) were higher in OLK than in OSCC, whereas infiltration of activated CD4 T cells (*P* = 0.001), T follicular helper cells (*P* = 0.026), M0 macrophages (*P* < 0.001), M1 macrophages (*P* < 0.001), and activated MCs (*P* = 0.030) were higher in OSCC than in OLK (Fig. 1A). The GSE85195 dataset contains 15 cases of OLK and 34 cases of OSCC, and the GSE30784 dataset contains 17 cases of OLK and 167 cases of OSCC. We also performed CIBERSORT immune cell infiltration analysis in these datasets. In the GSE85195 dataset (Fig. 1B), the infiltration of resting CD4 memory T cells (*P* = 0.003), monocytes (*P* < 0.001), M2 macrophages (*P* < 0.001), resting DCs (*P* < 0.001), and resting MCs (*P* < 0.001) were higher in OLK than OSCC, whereas the infiltration of M0 macrophages (*P* < 0.001), M1 macrophages (*P* = 0.002), activated DCs (*P* < 0.001), and activated MCs (*P* < 0.001) were higher in OSSC than in OLK. In GSE30784 dataset (Fig. 1C), the infiltration of regulatory T cells (Treg; *P* < 0.001), monocytes (*P* = 0.004), M2 macrophages (*P* = 0.004), resting DCs (*P* = 0.002), and resting MCs (*P* < 0.001) were higher in OLK than OSCC, and the infiltration of M0 macrophages (*P* < 0.001), activated MCs (*P* = 0.016), eosinophils (*P* = 0.033), and neutrophils (*P* = 0.019) were higher in OSSC than in OLK. The combination of the three datasets unveiled higher infiltration of monocytes, M2 macrophages, resting DCs, and resting MCs in OLK compared with OSCC. Conversely, OSCC showed higher infiltration of M0 macrophages and activated MCs compared with OLK. The differences observed in resting and activated MCs between oral precancer and oral cancer imply the role of MC activation in OSCC progression.

### Increased levels of activated MCs associated with development and poor prognosis of oral cancer

**Figure 2:**
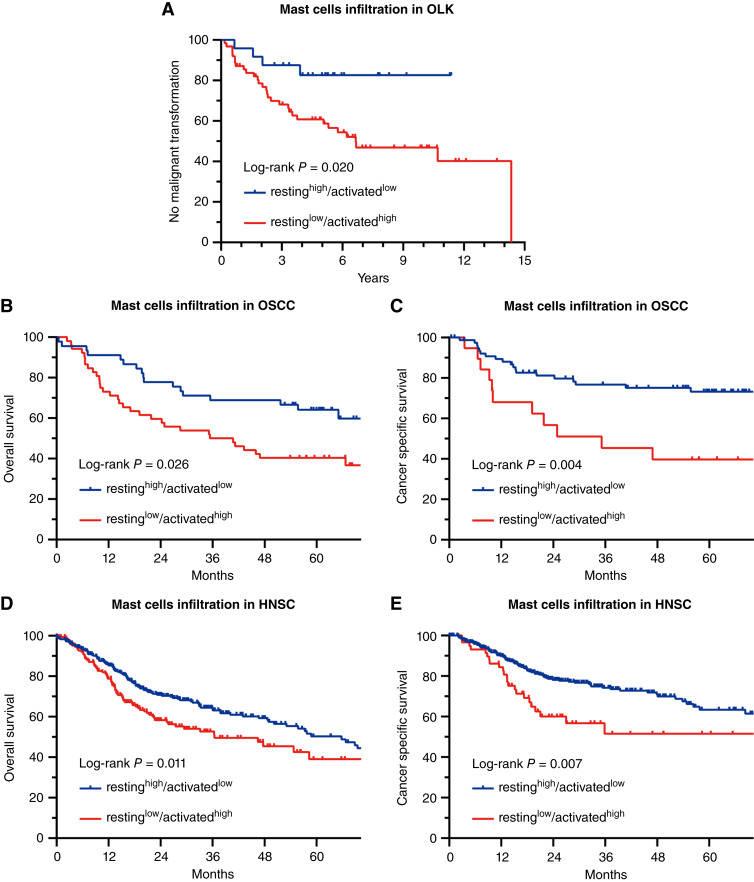
**A,** Cox regression analysis of an independent OLK dataset GSE26549 showed that the conversion of the resting to activated MC infiltration increased the risk of malignant transformation to OSCC. **B,** Cox regression analysis of the OSCC dataset GSE41613 showed that increased activated MC infiltration was associated with poor overall survival and **C,** cancer-specific survival in OSCC. Cox regression analysis of the TCGA HNSC cohort showed that increased activated MC infiltration was associated with **D,** poorer overall survival and **E,** cancer-specific survival in HNSC.

Cox regression analysis of an independent OLK dataset GSE26549 showed that resting MC infiltration was associated with a decreased risk of malignant transformation into OSCC [HR = 0.003, 95% confidence interval (CI): 0.000 to 0.666, *P* = 0.035] and activated MC infiltration was associated with an increased risk of malignant transformation to OSCC (HR = 4.213E+34, 95% CI: 1.220E+11–1.454E+58, *P* = 0.004). The conversion of the resting to activated MC infiltration increased the risk of malignant transformation to OSCC (*P* = 0.020), as shown by KM curve analysis (Fig. 2A). The cutoff scores of the MC populations were calculated based on the standardized log-rank statistics. Cox regression analysis of the OSCC dataset GSE41613 showed that resting MC infiltration was associated with better overall survival of OSCC (HR, < 0.001; 95% CI, 0.000–0.890; *P* = 0.047), and activated MC infiltration was associated with poor cancer-specific survival (HR, 299.578; 95% CI, 2.821–3.181E+4; *P* = 0.017). Increased activated MC infiltration was associated with poor overall survival (*P* = 0.026) and cancer-specific survival (*P* = 0.004) in OSCC as shown by KM curve analysis (Fig. 2B, C). Cox regression analysis of the TCGA HNSC cohort showed that resting MC infiltration was associated with better overall survival (HR, 0.005; 95% CI, 0.000–0.723; *P* = 0.037), whereas activated MC infiltration was associated with poor overall cancer survival (HR, 31.273; 95% CI, 1.432–683.089; *P* = 0.029). Resting MC infiltration was associated with better cancer-specific survival (HR < 0.001; 95% CI, 0.000–0.447; *P* = 0.029), and activated MC infiltration was associated with poor cancer-specific survival (HR, 161.043; 95% CI, 2.927–8.860E+3; *P* = 0.013). Increased activated MC infiltration was associated with poorer overall survival (*P* = 0.011) and cancer-specific survival (*P* = 0.007) in HNSC as shown by KM curve analysis (Fig. 2D and E).

### Correlations of immune cell infiltration in oral precancer and oral cancer

**Figure 3:**
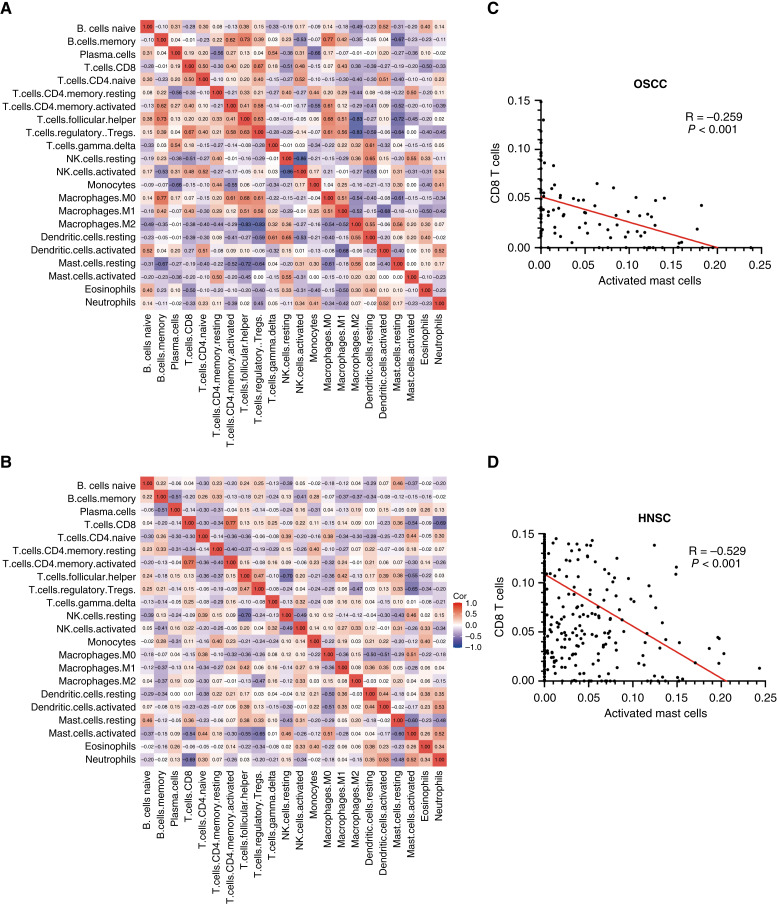
Correlations between the different types of immune cells in **A,** OLK and **B,** OSCC. MCs were negatively correlated with CD8 T cells in **C,** the GSE41613 dataset and **D,** the TCGA HNSC cohort.

We investigated potential relationships between different immune cell types in oral precancer and oral cancer by assessing correlations between the different types of immune cells. The levels of certain immune cell infiltrations are shown to correlate with each other in Fig. 3. In OLK samples (Fig. 3A), the infiltration of resting MCs was negatively correlated with B memory cells (*R* = −0.67), T follicular helper cells (*R* = −0.72), Tregs (*R* = −0.64), and M0 macrophages (*R* = −0.61). Moreover, the infiltration of activated MCs was negatively correlated with CD8 T cells (*R* = −0.54), T follicular helper cells (*R* = −0.55), and Tregs (*R* = −0.65), but positively correlated with resting NK cells (*R* = 0.46), M0 macrophages (*R* = 0.51), and neutrophils (*R* = 0.52) in OSCC samples (Fig. 3B). CD8 T cells are known to be the most important anti-tumor immune cells as they recognize antigens presented by tumor cells and kill these cells by releasing pro-inflammatory cytokines and cytolytic particles (9). It was speculated that activated MCs may crosstalk with CD8 T cells. The relationship between activated MCs and CD8 T cells in OSCC dataset GSE41613 and the TCGA HNSC cohort was investigated, and it was discovered that activated MCs were negatively correlated with CD8 T cells in GSE41613 dataset (*R* = −0.26; Fig. 3C) and the TCGA HNSC cohort (*R* = −0.53; Fig. 3D).

### MC promote tumor growth *in vivo*

**Figure 4:**
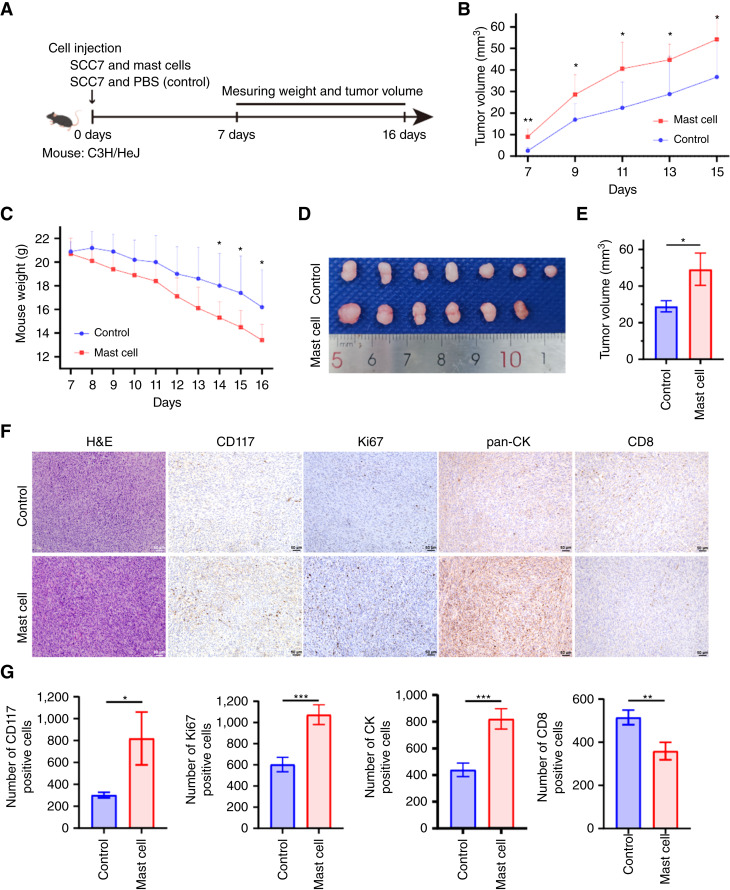
**A,** MCs promote tumor growth *in vivo*. Allograft model of the dorsal of tongue of the C3H / HeJ mouse using the squamous cell carcinoma cell line SCC7. **B,** A larger tumor volume was found in the experimental group compared with the control group at 7, 9, 11, 13, and 15 days. **C,** Weight of mice inoculated with a mixture of SCC7 and BMMCs was lower than that of mice inoculated with a mixture of SCC7 and PBS at 14 to 16 days. **D** and **E,** Mice inoculated with a mixture of SCC7 tumor cells and BMMCs showed a significantly higher volume. **F** and **G,** H&E staining identified the development of OSCC, and more CD117 (marker of MCs, *P* = 0.048), Ki67 (*P* < 0.001), and pan-CK (*P* < 0.001) positive cells found in the group inoculated with a mixture of SCC7 and BMMCs, whereas more CD8 positive cells were found in the control group. *, *P* < 0.05; ***, *P* < 0.001.

To explore the effect of MCs on OSCC *in vivo*, we constructed an allograft model of the dorsal of the tongue of the C3H/HeJ mouse using the squamous cell carcinoma cell line SCC7 (Fig. 4A). After inoculation with a mixture of SCC7 tumor cells and BMMCs (experimental group) or PBS (control group), a larger tumor volume was found in the experimental group compared with the control group at 7, 9, 11, 13, and 15 days (*P* < 0.05; Fig. 4B). Although there was no significant difference in weight between the two groups at 7 to 13 days, the weight of mice inoculated with a mixture of SCC7 and BMMCs was lower than that of mice inoculated with a mixture of SCC7 and PBS at 14 to 16 days (*P* < 0.05; Fig. 4C). All mice were sacrificed at the 17 days except 1 mouse of experimental group died before 17 days. The mice inoculated with a mixture of SCC7 tumor cells and BMMCs showed a significantly higher tumor volume (Fig. 4D and E). The H&E staining identified the development of OSCC, and more CD117 (a marker of MCs, *P* = 0.048), Ki67 (*P* < 0.001), and pan-CK (*P* < 0.001) positive cells were found in the group inoculated with a mixture of SCC7 and BMMCs, whereas more CD8 positive cells were found in the control group (*P* = 0.006; Fig. 4F and G). The BMMCs significantly promote the growth and proliferation of OSCC *in vivo*.

### MC subtypes in oral precancer and oral cancer

**Figure 5:**
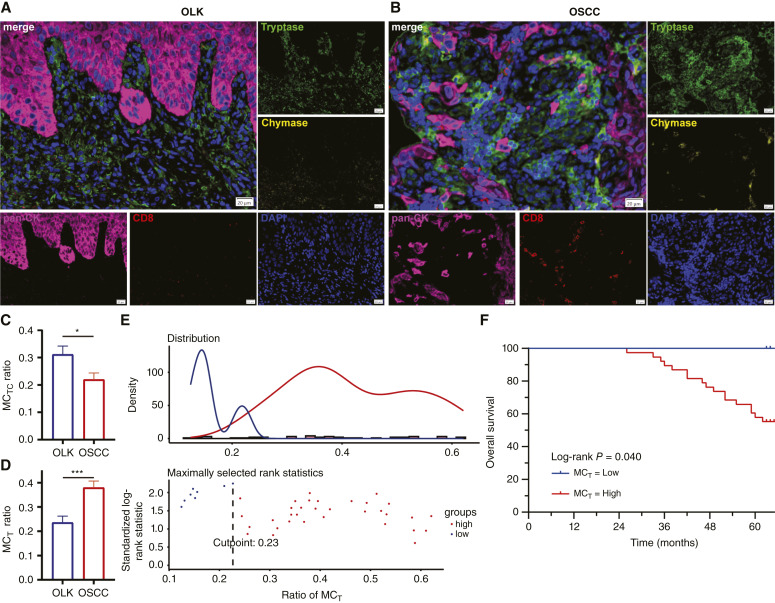
Multiplexed immunofluorescence staining in **A,** OLK and **B,** OSCC. **C,** Ratio of MC_TC_ in OLK was higher than that of MC_T_, **D,** whereas the ratio of MC_T_ was greater than that of MC_TC_ in OSCC. **E,** Ratio of MC_T_ was grouped based on the standardized log-rank statistic, and **F,** the KM curve survival analyses showed an association between MC_T_ and poor prognosis of OSCC. *, *P* < 0.05; ***, *P* < 0.001. DAPI, 4,6-diamidino-2-phenylindole.

Multiplex immunofluorescence staining for MCs identified a higher ratio of MC_TC_ in OLK, whereas a higher ratio of MC_T_ was found in OSCC. As shown in Fig. 5A–D, the ratio of MC_TC_ in OLK was higher than that of MC_T_ (*P* = 0.035), whereas the ratio of MC_T_ was greater than that of MC_TC_ in OSCC (*P* < 0.001). OLK had a higher amount of MC_TC_ than OSCC (*P* = 0.023; Fig. 5D). In contrast, OSCC had a higher quantity of MC_T_ than OLK (*P* < 0.001; Fig. 5E). We further investigate the association of MC subtypes with the prognosis of OLK and OSCC using Cox regression analysis in these formalin-fixed, paraffin-embedded samples. There was no association found between MC_TC_ (HR, 3.532; 95% CI, 0.572–21.812; *P* = 0.174) or MC_T_ (HR, 0.536; 95% CI, 0.060–4.823; *P* = 0.578) with the prognosis of OLK. There was no association between MC_TC_ (HR, 0.006; 95% CI, 0.114–2.101; *P* = 0.144) and OSCC prognosis. However, increased levels of MC_T_ (HR, 16.654; 95% CI, 1.246–222.561; *P* = 0.033) were associated with poorer overall survival in OSCC. As illustrated in Fig. 5E, the ratio of MC_T_ was categorized into two groups: a high ratio of MC_T_ group and a low ratio of MC_T_ group. The cutoff point was set at 0.23 based on the standardized log-rank statistic. Furthermore, the KM curve analysis revealed poor survival in the high MC_T_ group, as shown in Fig. 5F. The gradual increase in the distribution of MC_T_ from oral precancer to OSCC and its association with poor prognosis suggests that MC_T_ may play a major role in the development and progression of OSCC. Moreover, Supplementary Table S3 investigated the association between MC subtypes and clinical parameters in OLK and OSCC, revealing a higher ratio of MC_T_ in OSCC located in the palate compared with those found in the buccal and tongue (*P* = 0.022).

### Cellular spatial heterogeneity and interaction

We further analyzed the spatial distance between MCs, CD8^+^ cells, and cancer cells to clarify the spatial heterogeneity and interactions between cells (Supplementary Fig. S1A). The distributions of MC subtypes and CD8^+^ cells were investigated. It was discovered that the distances between MC_TC_ and CD8^+^ cells (all *P* < 0.01; Supplementary Fig. S1B), as well as between MC_T_ and CD8^+^ cells (all *P* < 0.001; Supplementary Fig. S1C), were lower in OSCC than in OLK within a radius of 40, 60, 80, and 100 µm, but there was no significant difference between them within a radius of 20 µm. These may imply that interactions between immune cells may occur more often in oral cancer compared with oral precancer. We then computed the mean distance between the two MC types within a radius of 20, 40, 60, 80, and 100 µm. As shown in Supplementary Fig. S1D, greater distances were found between MC_TC_ and cancer cells compared with MC_T_ and cancer cells in OSCC within a radius of 20, 40, 60, 80, and 100 µm (all *P* < 0.001). These suggest that the distribution of MC_T_ but not MC_TC_ is spatially closer to oral cancer, implying a potential interaction between MC_T_ and OSCC. The distances between MC_T_ and CD8^+^ cells were investigated in relation to the prognoses of OLK and OSCC. There was no association between the distances of MC_T_ and CD8^+^ cells and OLK prognosis. However, the greater distance between MC_T_ and CD8^+^ cells within a radius of 20 µm was associated with poor survival of OSCC (HR, 3.613; 95% CI, 1.172–11.144; *P* = 0.025). As illustrated in Supplementary Fig. S1E, the distances between MC_T_ and CD8^+^ cells within a radius of 20 µm were grouped based on the standardized log-rank statistic, and the KM curve survival analyses are demonstrated in Supplementary Fig. S1F.

### MC_T_ is an effective diagnostic/prognostic marker for OSCC

**Figure 6:**
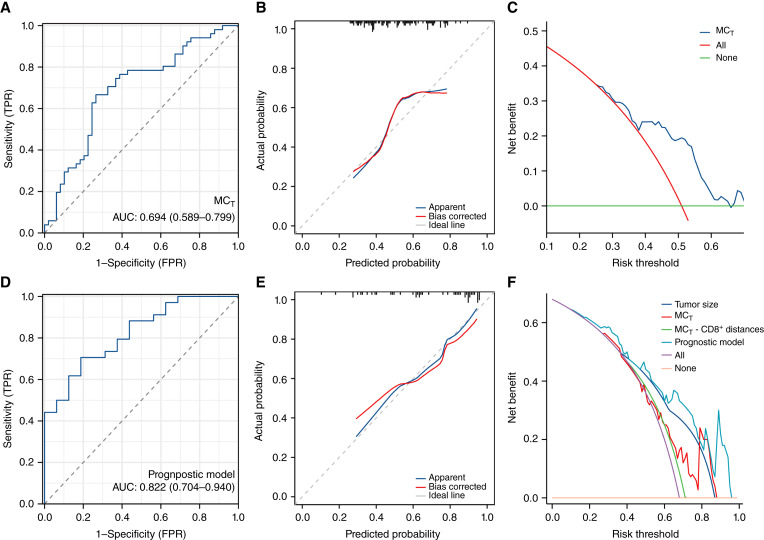
**A,** ROC curve indicated that the diagnostic AUC of MC_T_ was 0.694. **B,** Calibration curve showed that the predicted line was close to the reference line. **C,** Decision curve analyses demonstrated that except for a small range of low thresholds, the diagnostic model showed higher benefits compared with diagnosing all patients with OSCC or diagnosing none with OSCC. **D,** ROC curve indicated an AUC of 0.822 for prognostic evaluation. **E,** Calibration curve showed that the predicted line matched well with the reference line. **F,** Decision curve analyses demonstrated that except for a small range of low thresholds, the prognostic model and each of the prognostic markers showed higher benefits compared with predicting all patients with OSCC or predicting none with OSCC.

As shown in Supplementary Table S4, univariate logistic regression analyses showed a significant difference in MC_TC_ (OR = 0.095, 95% CI, 0.012 to 0.757; *P* = 0.026) and MC_T_ (OR = 54.300; 95% CI, 5.439, 542.082; *P* = 0.001) between OLK and OSCC. However, multivariate analysis demonstrated that only MC_T_ could serve as a diagnostic marker for OSCC (OR = 35.439; 95% CI, 3.080, 407.748; *P* = 0.004). The ROC curve indicated that the diagnostic AUC of MC_T_ was 0.694 (95% CI, 0.589–0.799; Fig. 6A). The calibration curve and decision curve analysis confirmed the accuracy and diagnostic potential of MC_T_ for clinical application (Fig. 6B and C). As shown in Supplementary Table S5, univariate and multivariate Cox regression analyses showed that the tumor size (*P* = 0.009), MC_T_ (*P* = 0.050), and the distances between MC_T_ and CD8^+^ cells within a radius of 20 µm (*P* = 0.010) were significantly associated with the prognosis of OSCC. These three markers were used to construct a prognostic model for 5-year survival of OSCC. The ROC curve indicated an AUC of 0.822 (95% CI, 0.704–0.940; Fig. 6D) for prognostic evaluation. Moreover, the calibration curve showed that the predicted line matched well with the reference line (Fig. 6E). Furthermore, the decision curve analyses demonstrated that except for a small range of low threshold, the prognostic model and each of the prognostic markers showed higher benefit than predicting all patients with OSCC or predicting none with OSCC (Fig. 6F).

## Discussion

MC is widely distributed, primarily at the interface between the host and the external environment (27). It has been implicated in the pathogenesis of allergic and autoimmune disorders and was involved in the IgE-mediated type I immune hypersensitivity response (13, 28). MCs were also present in the tumor microenvironment of various cancers and were suspected to be the infiltrating cells at the early stage during the development of cancers (29). This study indicates that MCs might contribute to the malignant transformation of oral precancer into oral cancer and interfere with CD8^+^ cells during OSCC progression. MC_T_ plays a crucial role in this process and may serve as a biomarker for OSCC diagnosis and prognosis.

Although PD1-based immunotherapy has been approved for clinical use in OSCC, the objective response rate is less than 20% (12). TME is a crucial factor in cancer progression and clinical outcomes for patients with OSCC. Therefore, it is necessary to develop novel immunotherapies that consider all key players in the complex TME to enhance the responses to immune checkpoint blockers (30). Improved insight into the biology of OSCC, especially the interactions between the immune cells of TME, could lead to the identification of new biomarkers for patient stratification in clinical decision-making and also aid in the development of innovative therapeutic approaches (9). Rojas and colleagues (31) found that the number of MC in lip SCC was higher than in normal lip mucosa. Similarly, Freitas and colleagues (32) reported that the density of MC_T_ was higher in OSCC than in actinic cheilitis and was positively correlated with the expression of MMP9. Kurihara-Shimomura and colleagues (33) identified that MC may induce activation and secretion of melanoma inhibitory activity genes and transport and Golgi organization protein 1 in OSCC. However, the role of the immune infiltration of MCs in the TME and prognosis of OSCC has not yet been clarified.

We found that activated MCs not only participate in the malignant transformation of oral precancer but also play an important role in OSCC progression. The increased infiltration of activated MCs is associated with development and poor survival in OSCC. The MC_T_ subtypes seem to play a prominent role in this process. The MC_T_ could be used not only as a biomarker to identify malignant transformation of oral precancer but also to identify poor prognosis in OSCC. These findings may enhance the insight of MCs in human cancer. MCs express vascular endothelial growth factor, which might play a role in the angiogenesis within the development of cancers (27). The c-Kit/stem cell factor axis is a significant MC-dependent pathway that affects MC development, migration, and activation (34). Stem cell factor can induce MC infiltration and activation, thereby exacerbating inflammation and immunosuppression in the tumor microenvironment (34). MC also releases proteases, including tryptase and chymase, which activate matrix metalloproteinases and degrade the extracellular matrix surrounding the cancer cells, promoting tumor growth, angiogenesis, and metastasis (29). Melillo and colleagues (35) found that histamines and chemokines mediate the promotion of growth and invasiveness of thyroid cancer by MC. Strouch and colleagues (36) reported that MC increases cancer cell proliferation and invasion in an MMP-dependent manner. The phenotype and function of MCs, as well as the extent of mediator production and release, vary depending on the stage of MC development and exposure to environmental inflammatory mediators (13, 34). Therefore, the impact of MCs on tumorigenesis may depend on various factors such as the type of tumor and the activation status of mast cells (13).

Moreover, we identified potential crosstalk between MC and CD8^+^ T cells using RNA-seq and histologic experiments. Across multiple datasets, activated MC and CD8^+^ T cells showed a significant negative correlation in OSCC samples. Additionally, both MC subtypes were significantly closer to CD8^+^ cells in OSCC than in oral precancer on the spatial distance analysis, which might be suggesting enhanced immune interactions in the TME after malignant transformation from oral precancer to OSCC. Furthermore, there was a significant correlation between poor prognosis in OSCC and the spatial distance of MC_T_ from CD8^+^ cells. CD8^+^ T cells are potent anticancer immune cells and are correlated with the efficacy of immunotherapy (37, 38). Therefore, our results suggested that MC infiltration, particularly the MC_T_ subtype, might interfere with CD8^+^ T cells and take part in the occurrence and development of OSCC. Eissmann and colleagues (39) discovered that IL33 activates MCs, leading to a signaling cascade that is dependent on MCs and macrophages, promoting the growth of gastric cancer. Somasundaram and colleagues (14) identified that co-localization of FOXP3^+^ Tregs and MCs in melanoma was correlated with resistance to anti-PD1 therapy and the efficacy of anti-PD1 therapy was improved by depleting MC using the MC inhibitors sunitinib and imatinib. In the future, it could be possible to develop targeted therapies against MC, which might enhance the immunotherapeutic efficacy of cancer.

However, there were limitations in this study. First, we have not identified the mechanisms of the interactions between MC, oral cancer cells, and CD8^+^ T cells, which may be related to the cytokines and chemokines secreted by MC (40). Second, in order for MC_T_ to become a clinically available diagnostic and prognostic biomarker for OSCC, it still needs to be validated in multicentre and prospective trials. Even so, our study provides new insights into MCs for decoding the cancer IME in OSCC, which could contribute to the development of innovative immunotherapies.

## Conclusion

This study found that activated MC infiltration is associated with the premalignant transformation and progression of oral cancer. MCs may influence the tumor IME in OSCC through interaction with CD8^+^ T cells. Additionally, the MC subtype MC_T_ may serve as a potential diagnostic and prognostic biomarker for OSCC. Developing immunotherapies targeting MCs may help improve clinical outcomes in OSCC.

## Supplementary Material

Supplementary Tables Supplementary Table 1. Baseline data of oral leukoplakia and oral squamous cell carcinoma samples for RNA sequencing. Supplementary Table 2. Baseline data of oral leukoplakia and oral squamous cell carcinoma samples for immunofluorescence staining. Supplementary Table 3. The association between mast cell subtypes and clinical parameters in oral leukoplakia and oral squamous cell carcinoma. Supplementary Table 4. The logistic regression analyses of potential diagnostic markers for oral leukoplakia and oral squamous cell carcinoma. Supplementary Table 5. The Cox regression analyses of potential prognostic markers for oral squamous cell carcinoma.

Supplementary Figure 1 Supplementary Figure 1. The spatial distance between mast cells, CD8+ cells and cancer cells (A). The distances between MCTC and CD8+ cells (B), as well as between MCT and CD8+ cells (C), were lower in OSCC than in OLK within a radius of 40µm, 60µm, 80µm, and 100µm, but there is no significant difference between them within a radius of 20µm. The greater distances were found between MCTC and cancer cells compared to MCT and cancer cells in OSCC within a radius of 20µm, 40µm, 60µm, 80µm, and 100µm (D). The distances between MCT and CD8+ cells within a radius of 20µm were grouped based on the standardized log-rank statistic (E), and the KM curve survival analyses showed that the greater distance between MCT and CD8+ cells within a radius of 20µm was associated with poor survival of OSCC (F). *, P < 0.05; **, P < 0.01; ***, P < 0.001; ns, not significant.
