# Supplementary material for: Mast Cell Infiltration and Subtype Promote Malignant Transformation of Oral Precancer and Progression of Oral Cancer
Source: Cancer Res Commun. 2024 Aug 22;4(8):2203–14. doi: 10.1158/2767-9764.CRC-24-0169 (PMC11339667; doi:10.1158/2767-9764.CRC-24-0169)
Supplement: Supplementary Tables — Supplementary Table 1. Baseline data of oral leukoplakia and oral squamous cell carcinoma samples for RNA sequencing. Supplementary Table 2. Baseline data of oral leukoplakia and oral squamous cell carcinoma samples for immunofluorescence staining. Supplementary Table 3. The association between mast cell subtypes and clinical parameters in oral leukoplakia and oral squamous cell carcinoma. Supplementary Table 4. The logistic regression analyses of potential diagnostic markers for oral leukoplakia and oral squamous cell carcinoma. Supplementary Table 5. The Cox regression analyses of potential prognostic markers for oral squamous cell carcinoma. [file crc-24-0169_supplementary_tables_suppst.docx]

**Mast cell infiltration and subtype promote malignant transformation of oral precancer and progression of oral cancer**

**Supplementary Table 1.** Baseline data of oral leukoplakia and oral squamous cell carcinoma samples for RNA sequencing

| Variables | Oral leukoplakia | Oral squamous cell carcinoma |
| --- | --- | --- |
| Age | 62.3 ± 6.2 | 66.3 ± 11.2 |
| Sex |  |  |
| Male | 3 (27.3%) | 9 (45.0%) |
| Female | 8 (72.7%) | 11 (55.0%) |
| Site |  |  |
| Buccal | 7 (63.6%) | 7 (35.0%) |
| Tongue | 4 (36.4%) | 6 (30.0%) |
| Gingiva | 0 (0.0%) | 7 (35.0%) |
| Tobacco smoking history |  |  |
| No | 11 (100.0%) | 17 (85.0%) |
| Yes | 0 (0.0%) | 3 (15.0%) |
| Alcohol consumption history |  |  |
| No | 11 (100.0%) | 15 (75.0%) |
| Yes | 0 (0.0%) | 5 (25.0%) |
| Epithelial dysplasia |  |  |
| Mild dysplasia | 7 (63.6%) | NA |
| Moderate dysplasia | 4 (36.4%) | NA |
| Tumor grade |  |  |
| Grade I | NA | 12 (60.0%) |
| Grade II | NA | 8 (40.0%) |

**Supplementary Table 2.** Baseline data of oral leukoplakia and oral squamous cell carcinoma samples for immunofluorescence staining

| Variables | Oral leukoplakia | Oral squamous cell carcinoma |
| --- | --- | --- |
| Age |  |  |
| < 60 | 30 (60.0%) | 23 (46.0%) |
| ≥ 60 | 20 (40.0%) | 27 (54.0%) |
| Sex |  |  |
| Male | 25 (50.0%) | 34 (68.0%) |
| Female | 25 (50.0%) | 16 (32.0%) |
| Site |  |  |
| Buccal | 8 (16.0%) | 10 (20.0%) |
| Tongue | 37 (74.0%) | 19 (38.0%) |
| Gingiva | 4 (8.0%) | 13 (26.0%) |
| Lip | 1 (2.0%) | 4 (8.0%) |
| Palate | 0 (0.0%) | 4 (8.0%) |
| Tobacco smoking history |  |  |
| No | 37 (74.0%) | 34 (68.0%) |
| Yes | 13 (26.0%) | 16 (32.0%) |
| Alcohol consumption history |  |  |
| No | 37 (74.0%) | 34 (68.0%) |
| Yes | 13 (26.0%) | 16 (32.0%) |
| Epithelial dysplasia |  |  |
| Mild dysplasia | 28 (56.0%) | NA |
| Moderate dysplasia | 22 (44.0%) | NA |
| Tumor grade |  |  |
| Grade I | NA | 24 (48.0%) |
| Grade II | NA | 26 (52.0%) |
| Tumor size |  |  |
| T1 | NA | 16 |
| T2 | NA | 23 |
| T3 & T4 | NA | 11 |
| Node stage |  |  |
| N0 | NA | 36 |
| N1 | NA | 6 |
| N2 | NA | 8 |
| Metastasis stage |  |  |
| M0 | NA | 50 |
| Tumor stage |  |  |
| I | NA | 14 |
| II | NA | 15 |
| III | NA | 10 |
| IV | NA | 11 |

**Supplementary Table 3.** The association between mast cell subtypes and clinical parameters in oral leukoplakia and oral squamous cell carcinoma

| Variables | Oral leukoplakia | | | | Oral squamous cell carcinoma | | | | |
| --- | --- | --- | --- | --- | --- | --- | --- | --- | --- |
|  | MC_TC_ | *P* value | MC_T_ | *P* value | MC_TC_ | *P* value | MC_T_ | *P* value | |
| Age |  |  |  |  |  |  |  |  | |
| < 60 | 0.27 ± 0.19 | 0.116 | 0.26 ± 0.20 | 0.247 | 0.26 ± 0.20 | 0.153 | 0.32 ± 0.19 | 0.053 | |
| ≥ 60 | 0.38 ± 0.26 |  | 0.20 ± 0.16 |  | 0.19 ± 0.14 |  | 0.43 ± 0.18 |  | |
| Sex |  |  |  |  |  |  |  |  | |
| Male | 0.35 ± 0.24 | 0.206 | 0.22 ± 0.19 | 0.465 | 0.19 ± 0.18 | 0.060 | 0.37 ± 0.18 | 0.648 | |
| Female | 0.27 ±0.19 |  | 0.26 ± 0.18 |  | 0.29 ± 0.14 |  | 0.40 ± 0.22 |  | |
| Site |  |  |  |  |  |  |  |  | |
| Buccal | 0.32 ± 0.23 | 0.078 | 0.26 ± 0.22 | 0.757 | 0.21 ± 0.20 | 0.527 | 0.29 ± 0.18 | 0.022 | |
| Tongue | 0.29 ± 0.21 |  | 0.24 ± 0.18 |  | 0.20 ± 0.18 |  | 0.36 ± 0.20 |  | |
| Gingiva | 0.55 ± 0.22 |  | 0.20 ± 0.20 |  | 0.29 ± 0.17 |  | 0.38 ± 0.16 |  | |
| Lip | 0.11 |  | 0.06 |  | 0.16 ± 0.12 |  | 0.45 ± 0.13 |  | |
| Palate | NA | NA | NA | NA | 0.18 ± 0.08 |  | 0.65 ± 0.08 |  | |
| Tobacco smoking history | | | | | | | | |  |
| No | 0.31 ± 0.20 | 0.974 | 0.25 ± 0.18 | 0.405 | 0.25 ± 0.17 | 0.095 | 0.36 ± 0.17 | 0.218 | |
| Yes | 0.31 ± 0.29 |  | 0.20 ± 0.21 |  | 0.16 ± 0.16 |  | 0.43 ± 0.23 |  | |
| Alcohol consumption history | | | | | | | | |  |
| No | 0.31 ± 0.21 | 0.806 | 0.24 ± 0.18 | 0.966 | 0.24 ± 0.15 | 0.166 | 0.40 ± 0.19 | 0.300 | |
| Yes | 0.32 ± 0.25 |  | 0.24 ± 0.22 |  | 0.17 ± 0.21 |  | 0.34 ± 0.21 |  | |

**Supplementary Table 4.** The logistic regression analyses of potential diagnostic markers for oral leukoplakia and oral squamous cell carcinoma

| Variables | Univariate analyses | | Multivariate analyses | |
| --- | --- | --- | --- | --- |
|  | Odds ratio (95% CI) | *P* value | Odds ratio (95% CI) | *P* value |
| Age |  |  |  |  |
| < 60 | Reference |  |  |  |
| ≥ 60 | 1.761 (0.796, 3.893) | 0.162 |  |  |
| Sex |  |  |  |  |
| Male | Reference |  |  |  |
| Female | 0.471 (0.209, 1.061) | 0.069 |  |  |
| Site |  |  |  |  |
| Buccal | Reference |  |  |  |
| Tongue | 0.411 (0.139, 1.212) | 0.107 |  |  |
| Gingiva | 2.600 (0.606, 11.152) | 0.198 |  |  |
| Lip | 3.200 (0.296, 34.588) | 0.338 |  |  |
| Palate | NA |  |  |  |
| Tobacco smoking history |  |  |  |  |
| No | Reference |  |  |  |
| Yes | 1.339 (0.563, 3.189) | 0.509 |  |  |
| Alcohol consumption history |  |  |  |  |
| No | Reference |  |  |  |
| Yes | 1.339 (0.563, 3.189) | 0.509 |  |  |
| MC_TC_ ratio | 0.095 (0.012, 0.757) | 0.026 | 0.335 (0.033, 3.349) | 0.352 |
| MC_T_ ratio | 54.300 (5.439, 542.082) | 0.001 | 35.439 (3.080, 407.748) | 0.004 |

**Supplementary Table 5.** The Cox regression analyses of potential prognostic markers for oral squamous cell carcinoma

| Variables | Univariate analyses | | Multivariate analyses | |
| --- | --- | --- | --- | --- |
|  | Hazard ratio (95% CI) | *P* value | Hazard ratio (95% CI) | *P* value |
| Age |  |  |  |  |
| < 60 | Reference |  |  |  |
| ≥ 60 | 2.739 (0.984, 7.625) | 0.054 |  |  |
| Sex |  |  |  |  |
| Male | Reference |  |  |  |
| Female | 1.251 (0.462, 3.384) | 0.660 |  |  |
| Site |  |  |  |  |
| Buccal | Reference |  |  |  |
| Tongue | 1.068 (0.275, 4.158) | 0.924 |  |  |
| Gingiva | 1.433 (0.353, 5.809) | 0.615 |  |  |
| Lip | NA |  |  |  |
| Palate | 1.566 (0.261, 9.401) | 0.624 |  |  |
| Tobacco smoking history |  |  |  |  |
| No | Reference |  |  |  |
| Yes | 1.352 (0.536, 3.409) | 0.522 |  |  |
| Alcohol consumption history |  |  |  |  |
| No | Reference |  |  |  |
| Yes | 1.041 (0.404, 2.682) | 0.933 |  |  |
| Tumor grade |  |  |  |  |
| Grade I | Reference |  |  |  |
| Grade II | 2.216 (0.840, 5.848) | 0.108 |  |  |
| Tumor size |  |  |  |  |
| T1 | 0.413 (0.134, 1.271) | 0.123 | 0.185 (0.052, 0.660) | 0.009 |
| T2 | 0.238 (0.075, 0.757) | 0.015 | 0.180 (0.050, 0.648) | 0.009 |
| T3 & T4 | Reference |  | Reference |  |
| Node stage |  |  |  |  |
| N0 | Reference |  |  |  |
| N1 | 2.849 (0.950, 8.540) | 0.062 |  |  |
| N2 | 0.745 (0.166, 3.339) | 0.701 |  |  |
| Tumor stage |  |  |  |  |
| I | Reference |  |  |  |
| II | 0.328 (0.063, 1.701) | 0.184 |  |  |
| III | 2.165 (0.683, 6.857) | 0.189 |  |  |
| IV | 1.565 (0.447, 5.481) | 0.483 |  |  |
| MC_TC_ ratio | 0.114 (0.006, 2.101) | 0.144 |  |  |
| MC_T_ ratio | 16.654 (1.246, 222.561) | 0.033 | 20.270 (1.000, 410.785) | 0.050 |
| Distance between MC_T_ and CD8^+^ cells within a radius of 20µm | 3.613 (1.172, 11.144) | 0.025 | 5.457 (1.511, 19.707) | 0.010 |
