# Supplementary material for: Mast Cell Infiltration and Subtype Promote Malignant Transformation of Oral Precancer and Progression of Oral Cancer
Source: Cancer Res Commun. 2024 Aug 22;4(8):2203–14. doi: 10.1158/2767-9764.CRC-24-0169 (PMC11339667; doi:10.1158/2767-9764.CRC-24-0169)
Supplement: Supplementary Figure 1 — The spatial distance between mast cells, CD8+ cells and cancer cells (A). The distances between MCTC and CD8+ cells (B), as well as between MCT and CD8+ cells (C), were lower in OSCC than in OLK within a radius of 40µm, 60µm, 80µm, and 100µm, but there is no significant difference between them within a radius of 20µm. The greater distances were found between MCTC and cancer cells compared to MCT and cancer cells in OSCC within a radius of 20µm, 40µm, 60µm, 80µm, and 100µm (D). The distances between MCT and CD8+ cells within a radius of 20µm were grouped based on the standardized log-rank statistic (E), and the KM curve survival analyses showed that the greater distance between MCT and CD8+ cells within a radius of 20µm was associated with poor survival of OSCC (F). *, P < 0.05; **, P < 0.01; ***, P < 0.001; ns, not significant. [file crc-24-0169_supplementary_figure_1_suppsf.docx]

**Mast cell infiltration and subtype promote malignant transformation of oral precancer and progression of oral cancer**


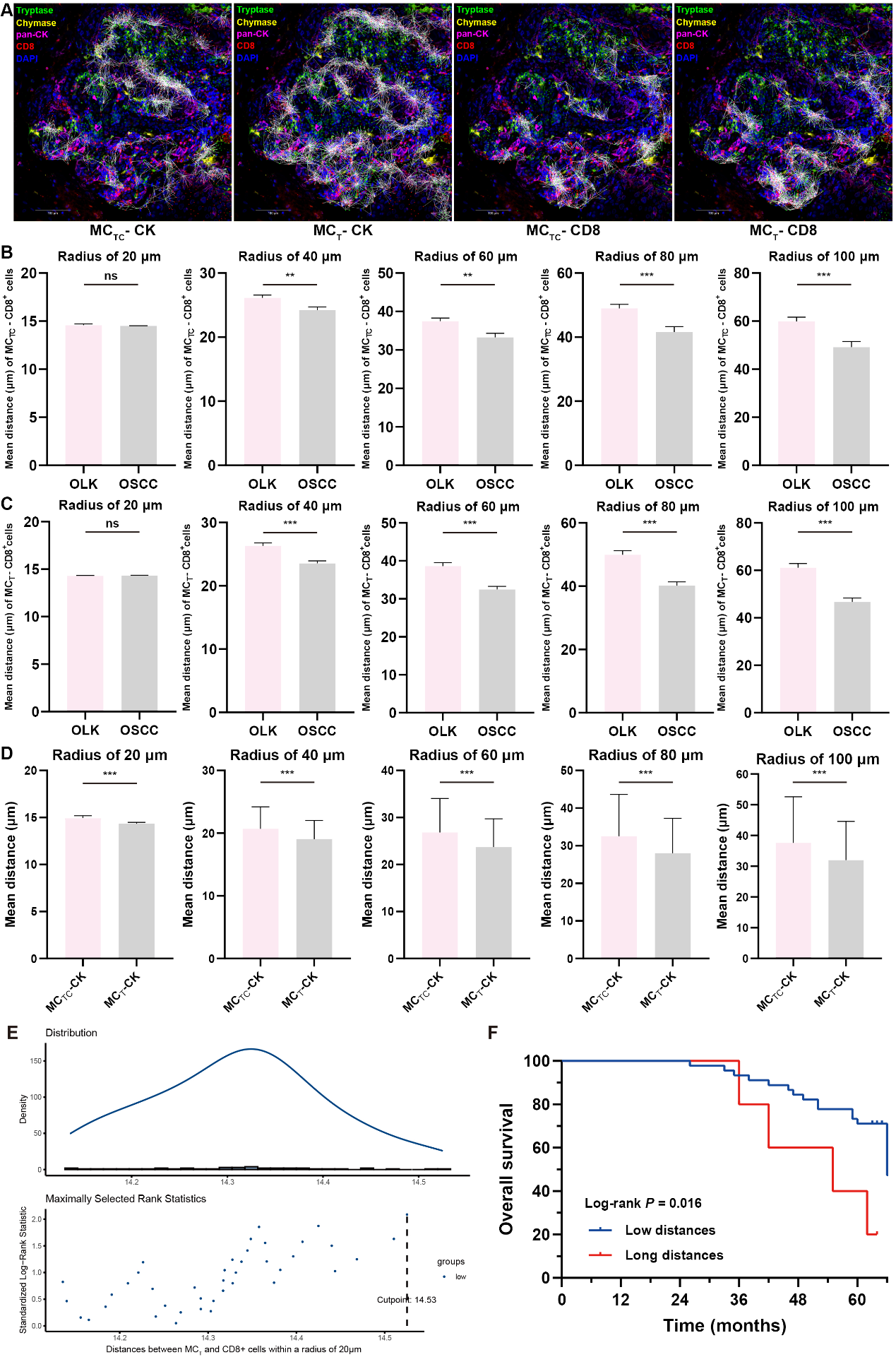


**Supplementary Figure 1.** The spatial distance between mast cells, CD8^+^ cells and cancer cells (A). The distances between MC_TC_ and CD8^+^ cells (B), as well as between MC_T_ and CD8^+^ cells (C), were lower in OSCC than in OLK within a radius of 40µm, 60µm, 80µm, and 100µm, but there is no significant difference between them within a radius of 20µm. The greater distances were found between MC_TC_ and cancer cells compared to MC_T_ and cancer cells in OSCC within a radius of 20µm, 40µm, 60µm, 80µm, and 100µm (D). The distances between MC_T_ and CD8^+^ cells within a radius of 20µm were grouped based on the standardized log-rank statistic (E), and the KM curve survival analyses showed that the greater distance between MC_T_ and CD8^+^ cells within a radius of 20µm was associated with poor survival of OSCC (F). ^*^, *P* < 0.05; ^**^, *P* < 0.01; ^***^, *P* < 0.001; ns, not significant.
